# Progressive Quadriparesis of a Toddler with a Posterior Cranial Fossa Arachnoid Cyst (AC): Illustrative Case Report and Narrative Literature Review

**DOI:** 10.3390/children11121463

**Published:** 2024-11-29

**Authors:** Thanos Vassilopoulos, Marianna Miliaraki, Christos Tsitsipanis, Konstantinos Ntotsikas, Nikolaos Chochlidakis, Dimitrios Karabetsos, Nikolaos Moustakis, Athanasios Theofanopoulos, Sofia Lazarioti, Vasilios Papastergiou, Georgia Kritikou, Andreas Yannopoulos

**Affiliations:** 1 School of Medicine, University of Crete, 71003 Heraklion, Crete, Greece; med4059@edu.med.uoc.gr; 2 Pediatric Intensive Care Unit, University Hospital of Heraklion, School of Medicine, University of Crete, Stavrakia, Voutes, 71003 Heraklion, Crete, Greece; 3 Neurosurgery Department, University Hospital of Heraklion, School of Medicine, University of Crete, 71003 Heraklion, Crete, Greece; ctsitsipanis@pagni.gr (C.T.);; 4 General Surgery Department, University Hospital of Heraklion, School of Medicine, University of Crete, 71003 Heraklion, Crete, Greece

**Keywords:** children, adult, arachnoid cysts, neurosurgery, posterior cranial fossa

## Abstract

Background/Objectives: Intracranial arachnoid cysts (ACs) may be congenital, primary, or secondary due to trauma. These cysts are benign, contain cerebrospinal fluid (CSF), and are classified based on location, size, and their clinical symptomatology. They are uncommon lesions in children, rarely leading to severe mass-effect neurological symptomatology. Methods: The present report describes a 30-month-old female presenting with quadriparesis. An emergency magnetic resonance imaging (MRI) study revealed the presence of a primary intracranial arachnoid cyst of the posterior cranial fossa, exerting significant pressure on the medulla oblongata and the cervical portion of the spinal cord, displacing them dorsally, with a remnant diameter of 2.5 mm. Results: This benign malformation located in a crucial area might have been complicated by severe neurological deterioration and required prompt intervention, so the patient underwent a suboccipital craniectomy in a sitting position, along with a neurosurgical procedure, which established a lasting communication channel between the cyst and the basal cisterns. This led to a favorable outcome. Conclusions: Up to the present report, postoperative complete resolution of quadriparesis secondary to a posterior cranial fossa arachnoid cyst has not been reported. At present, no therapeutic modality has been established as the definitive standard of care for pediatric ACs, and their management raises a great deal of controversy among neurosurgeons. The narrative literature review of the present study integrates the various perspectives regarding ACs and their possible treatment approaches that are currently available.

## 1. Introduction and Clinical Significance

Arachnoid cysts (ACs) are among the most common pathologies encountered by pediatric neurosurgeons, and they currently tend to come to clinical attention more frequently than in the past, due to the wide availability of diagnostic imaging studies [1]. Richard Bright first described patient cases with intracranial arachnoid cysts as early as 1831 [2,3]. The term “cyst” originates from the Greek word “kystis”, which describes a pouch-like cavity filled with fluid [4]. ACs are benign cystic malformations, which originate from splitting layers of the meninges, leading to mass-effect neurological signs through various pathophysiological mechanisms [5,6,7]. The frequency of ACs in children has been reported as high as 2.6%, although their incidence is even greater in the presence of certain congenital anomalies or genetic syndromes [8,9]. The underlying pathophysiology for these developmental cystic anomalies has not been entirely elucidated [9]. Based on the current literature, clinical manifestations of such cases are highly variable, and no therapeutic modality has been established as the definitive standard of care for pediatric arachnoid cysts [10,11,12,13]. Most asymptomatic intracranial arachnoid cysts are usually an incidental finding and can be monitored, but there is a small percentage of pediatric ACs that might require complex neurosurgical interventions [14].

The aim of this report is the presentation of a rare pediatric patient case with progressive quadriparesis due to a posterior cranial fossa arachnoid cyst extending to the upper cervical area. This patient underwent certain neurosurgical interventions, eventually leading to a favorable outcome. Moreover, the purpose of the narrative literature review in the present study is to integrate the various perspectives regarding ACs and their possible treatment approaches that are currently available.

## 2. Case Presentation

**Figure 1:**
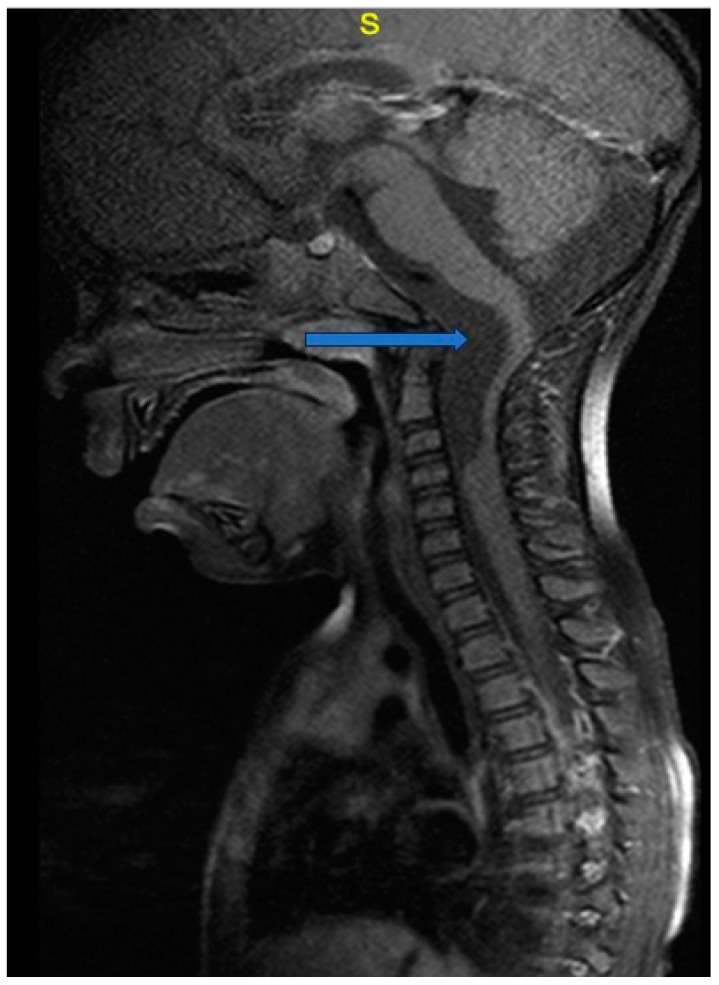
Preoperative magnetic resonance image: T1-weighted image, sagittal section. The arrow points to the hypointense lesion ventrally to the pons, medulla oblongata, and the superior cervical cord, representing the arachnoid cyst (AC).

**Figure 2:**
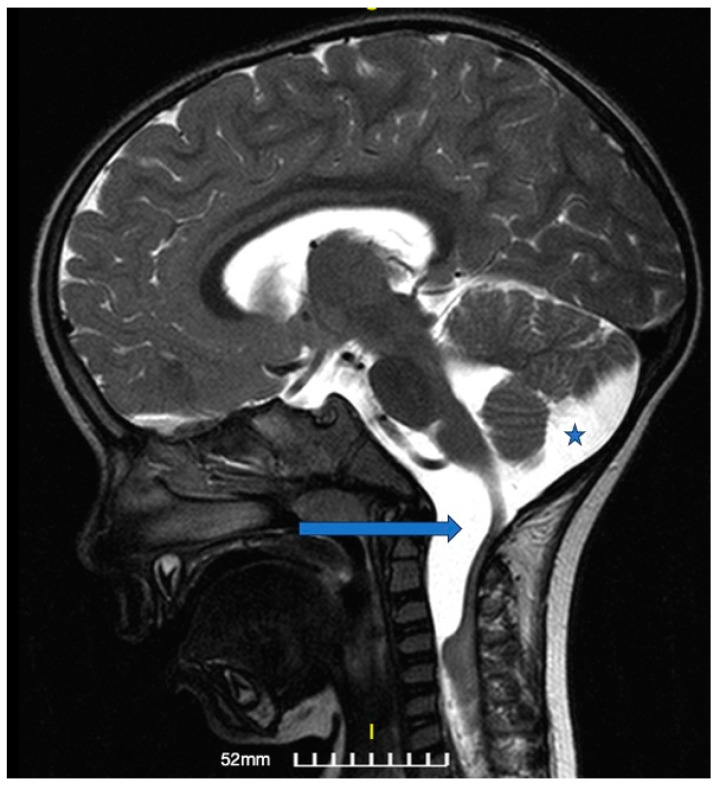
Preoperative magnetic resonance image: T2-weighted image, sagittal section. The arrow points to the hyperintense lesion ventrally to the pons, medulla oblongata, and the superior cervical cord, representing the arachnoid cyst (AC). The star is located at the large cisterna magna.

**Figure 3:**
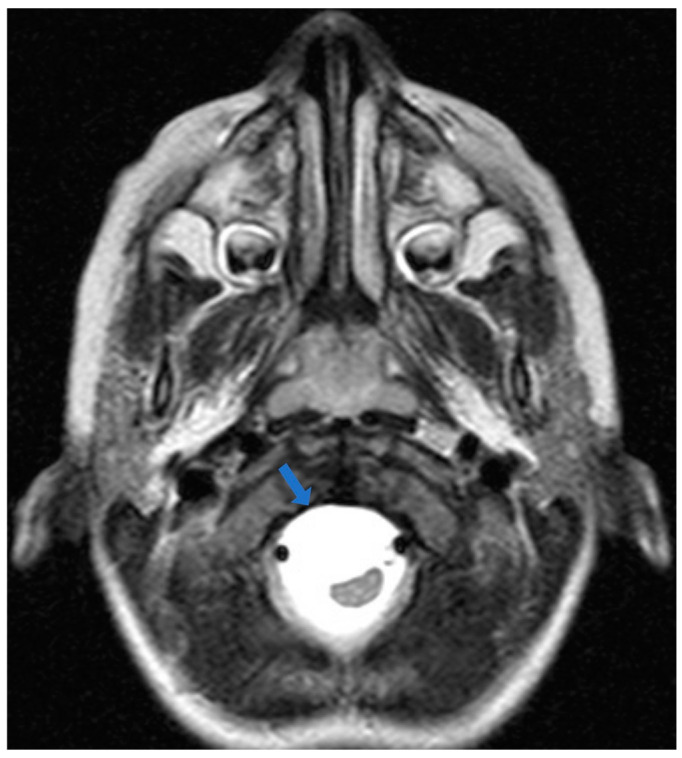
Preoperative magnetic resonance image: T2-weighted image, transverse section. The arrow points to the hyperintense lesion ventrally to the medulla oblongata, representing the arachnoid cyst (AC). The medulla has been dislocated posterolateral towards the left side.

**Figure 4:**
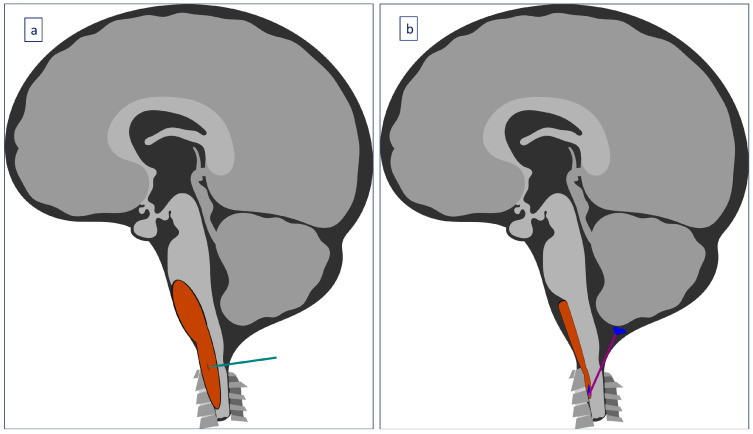
Illustration of the neurosurgical procedure performed in this pediatric case: (**a**) The patient underwent a suboccipital craniectomy in the sitting position, and the dura was opened, giving access to the cyst wall. The lower part of the cyst was fenestrated. (**b**) An additional small drainage tube established a permanent communication between the cyst remnant and the basal cisterns, in order to prevent future revision surgical interventions. Copyright: Benjamin Konstantinos Papadakis. Published with permission.

A 30-month-old female was transferred to the emergency department (ED) due to progressively worsening quadriparesis. A two-week history of torticollis was reported. Tonsillitis was also present, having been treated with antibiotics. Upon medical examination, the patient presented with left-sided hemiplegia and right-sided hemiparesis, with no concurrent sensory neurological signs. An emergency magnetic resonance imaging (MRI) study was performed, revealing the presence of an elongated (4.4 cm in length, 1.6 × 1 cm in diameter), thin-walled intradural, extramedullary arachnoid cyst in the medulla oblongata cistern, extending caudally to the level of the C4–C5 vertebrae (Figure 1, Figure 2 and Figure 3). This AC exerted significant pressure mainly on the premedullary cistern and the cervical portion of the spinal cord, displacing them dorsally, with a remnant diameter of 2.5 mm. The vertebral arteries were also displaced, yet patent. Medullary edema of the cord’s dorsal side was also present. Based on these findings, the decision for an urgent surgical procedure was made, so the patient underwent a suboccipital craniectomy in the sitting position. The posterior arch of the C1 vertebra was found to be hypoplastic, so it was resected to provide surgical space. The dura was subsequently opened and gave access to the cyst wall located under the C1 and C2 nerve roots, while microsurgical techniques permitted partial cyst excision. The cyst wall was meticulously opened as far as possible laterally and caudally, and a small-diameter drainage tube was placed within the cyst remnant, in case of adhesions and recurrences of the AC occurring in the future. Given the presence of the cisterna magna within the surgical field, this step established a lasting communication channel between the cyst and the basal cisterns. This tube did not serve as a drainage tube or a shunt device, given the extended fenestration of the AC wall. This step was taken in order to ensure that the condition would not relapse, and to prevent future revision surgical interventions (Figure 4). The dural closure was executed through the employment of a fascia lata patch, procured from the corresponding patient’s left femoral region. Following the surgical procedure, the patient was transferred to the pediatric intensive care unit (ICU) for postoperative care.

## 3. Results

**Figure 5:**
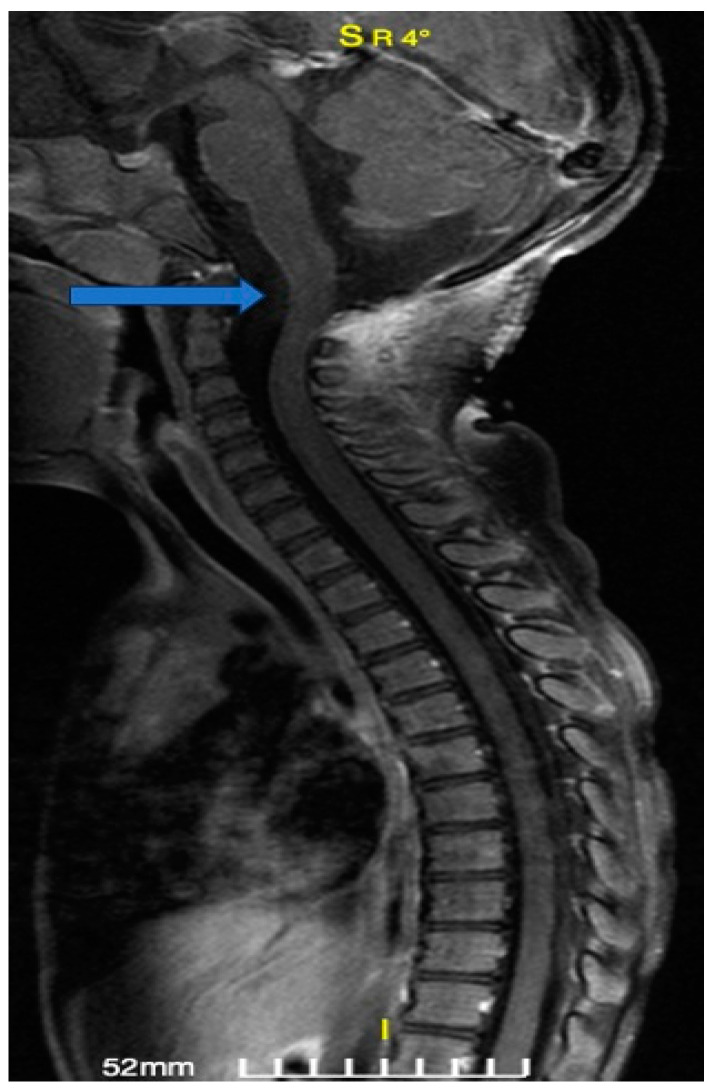
Postoperative magnetic resonance image at 3-month follow-up: T1-weighted image, sagittal section. The arrow points to the residual arachnoid cyst (AC). The neuronal structures are shown decompressed.

**Figure 6:**
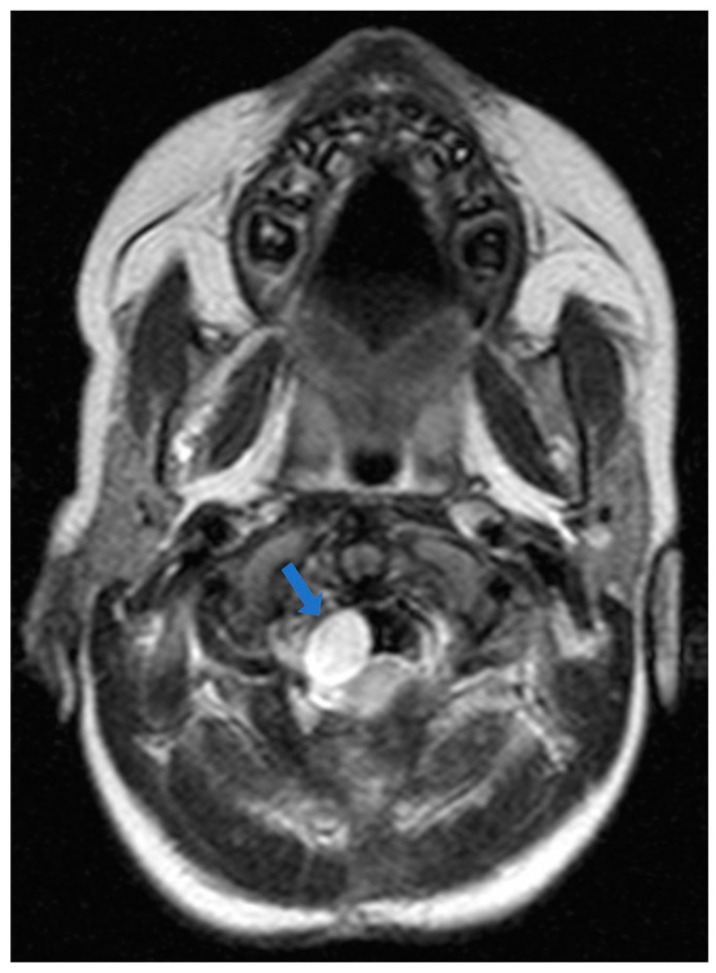
Postoperative magnetic resonance image at 12-month follow-up: T2-weighted image, axial section. The arrow points to the residual arachnoid cyst (AC). However, the neuronal structures are shown decompressed.

After 48 h of vigilant observation and monitoring, the patient demonstrated vital signs of stability and was safely transferred back to the ward. Significant progress in this patient’s physical condition had already been observed. Her right-sided muscle strength had already returned to normal, and apparent improvement in her left-sided hemiplegia was also noted. On the 7th postoperative day, the patient was able to ambulate autonomously (muscle strength of 5), thereby fulfilling the necessary criteria for discharge. In furtherance of ensuring adequate convalescence, the patient was scheduled to repeat a postoperative MRI at 3- and 12-month follow-up, which verified her stable clinical course (Figure 5 and Figure 6).

## 4. Discussion

The present report describes a rare case of a 30-month-old female presenting with quadriparesis due to a large, infratentorial arachnoid cyst, which had to be treated with quite challenging neurosurgical procedures. Up to the present report, no therapeutic modality has been established as the definitive standard of care for arachnoid cysts. Their management still raises a great deal of controversy among neurosurgeons, especially for pediatric patients [10]. Based on the current literature, clinical manifestations of similar cases of pediatric posterior cranial fossa ACs are highly variable, including severe neuropsychological impairment, cranial nerve palsies, hydrocephalus, or torticollis [11,12,13,15]. According to a large case series of pediatric patients with posterior fossa ACs, different neurosurgical approaches are usually chosen: most commonly microsurgical fenestration with or without cyst–peritoneal shunting, or endoscopic fenestration [16]. The narrative literature review of the present study highlights the importance of imaging in the diagnosis of primary or congenital symptomatic intracranial ACs and integrates the various perspectives regarding arachnoid cysts and their possible treatment approaches that are currently available.

### 4.1. Epidemiology

Arachnoid cysts (ACs) account for approximately 1% of all space-occupying, atraumatic intracranial mass lesions [17,18,19,20], with recent prevalence estimates of around 2.6% for children [21,22,23,24,25,26,27] and up to 1.7% for adults [28]. In addition, males are more likely to experience this condition [26,27]. Regarding the locations where ACs tend to occur more frequently, it is essential to note that certain areas have been identified as the most common regions for ACs to be found. The majority of ACs are supratentorial [19,29,30], with a clear dominance of ACs consistently found in the middle cranial fossa [2,7,27,31,32,33], although there are several reports of ACs located in the retrocerebellar region of the posterior cranial fossa [27,31,32,34]. These common localizations are most frequently associated with asymptomatic ACs, whereas ACs in less common regions tend to be symptomatic [28]. The left side of the intracranial space has also been reported to be implicated almost four times more frequently than the right side [4]. Some examples of such findings and their frequency, based on current studies, are shown in Table 1.

### 4.2. Etiology

Arachnoid cysts are likely idiopathic congenital lesions that occur due to abnormal splitting or duplication of the primitive arachnoid membrane during early embryonic development [20,26,30,40,42]. However, they can also occur due to other secondary causes, such as head injury or perinatal trauma, intracranial hemorrhage, inflammation, prematurity, and infection [6,20,30,42,43]. In addition, the possible association and coexistence between ACs and certain syndromes may explain a possible causative genetic mechanism [24,44]. Possible genetic syndromes connected to ACs that have been reported include Down syndrome or some other types of trisomies [35,36,38], schizencephaly [35,36,39], neurofibromatosis [35,36,38], mycopolysaccharidosis [35,36], autosomal-dominant polycystic kidney disease [19,35], Aicardi syndrome [35,38], glutaric aciduria type 1 [36,39], Marfan syndrome [28], or corpus callosum dysgenesis [38,39]. With regard to recent classifications based on AC localization, infratentorial ACs could possibly be located in the retrocerebellar space, intraventricular space, cerebellopontine angle, or in the quadrigeminal cistern region of the posterior cranial fossa, whereas supratentorial ACs are usually found in the Sylvian fissure, suprasellar region, cerebral convexity, interhemispheric fissure, intraventricular space, or in the quadrigeminal cistern region [28,45]. Another classification of ACs is based on the Galassi system, which takes into account both their location and size [28]. Spinal ACs are a rare cause of spinal cord compression, and they are most commonly extradural, arising dorsally to the spinal cord, and some rare cases of intradural or intramedullary ACs have been reported in the literature [46].

### 4.3. Pathophysiology

There is not a uniform approach when it comes to the pathogenesis of pediatric arachnoid cysts. As mentioned earlier, several studies support the mechanism of congenital splitting and duplication of arachnoid layers (endomeninx), a phenomenon that creates certain spaces filled with fluid [3,18,24,29,35,38,39]. Another possible pathophysiological event that has been proposed for some AC cases could be a malformation or defect of the two-layer fold membrane of Liliequist, leading to cystic dilation of adjacent cisterns and, thus, the creation of fluid-filled spaces [37]. Apart from maldevelopment, there are also some other theories suggesting how ACs may be created and increase in size in the long term. One reported theory is the valve mechanism theory, which suggests a unidirectional valve mechanism that drives fluid into the AC via pulsations [26,29,47,48,49]. Another possible mechanism could be the creation of an osmotic gradient, leading to fluid diffusion into the arachnoid cyst [26,35,36,48]. In addition, some studies support the theory of wall secretory properties, referring to specific cyst cells that are responsible for fluid production in the arachnoid cyst [3,23,29,36,47]. Moreover, venous agenesis or lobe agenesis may also explain a possible dysfunction of cerebrospinal fluid (CSF) drainage, contributing to fluid accumulation and expansion of an AC [24,36,47,48]. The only known risk factor for the enlargement of ACs is young age [23,35,39], although the majority of these lesions seem to remain stable over time [7,18,35,39,47].

### 4.4. Clinical Presentation

The clinical presentation of pediatric ACs can vary widely between patients. The majority of pediatric patients with ACs tend to be asymptomatic, and their cystic lesions are found incidentally during routine neuroimaging studies [22,24,29,35,47]. However, there are plenty of studies in the literature describing a wide spectrum of symptoms and signs associated with ACs. Symptomatic AC cases usually cause symptoms due to mass-effect or cyst-rupture phenomena [28]. Arachnoid cyst rupture might occur secondary to traumatic brain injury, but spontaneous rupture of ACs has very rarely been reported in children [50]. Nearly two-thirds of pediatric ACs are supratentorial, most commonly presenting with headache [51]. Arachnoid cysts of the posterior fossa are less frequent, and they usually present with craniomegaly, increased intracranial pressure (ICP), multiple cerebellar signs, or cervical spinal cord compression [36]. Moreover, a recent report describes a specific pattern of neurocognitive deficits in patients with posterior fossa cerebellar lesions, such as apathy, obsessive–compulsive traits, or dysphoria [52]. This study suggested that the presence of ACs during early life disrupts the maturation of distant temporal regions, through the phenomenon of developmental diaschisis, with an unfavorable impact on developmental social processes [52]. Spinal ACs are rare and usually affect the thoracic region of the spinal column, leading to backache, neurogenic bladder, and sensory or motor disturbances [28]. A list of symptoms most commonly associated with pediatric ACs and their frequency, based on the current literature, is shown in Table 2.

### 4.5. Assessment and Diagnosis

Regarding the diagnosis of ACs, it is easier to detect possible ACs in pediatric patients today, due to the growing availability of different neuroimaging modalities [19,22,25,29,35]. Intracranial ACs can be described as well-circumscribed and extra-axial cystic lesions in magnetic resonance imaging (MRI) and computed tomography (CT) [2,7,35,36,58]. More specifically, ACs are isointense to CSF on MRI sequences and are not contrast-enhanced [2,7,29,35,36,58]. Special pulse sequences are usually employed for the diagnosis of most thin-walled ACs, while a comprehensive MRI protocol is often the preferred modality for assessing ACs, including T1-weighted images for overall anatomy, T2-weighted images for localization, T2-fluid-attenuated inversion recovery (FLAIR) for surrounding edema, diffusion-weighted imaging (DWI) for distinguishing the cyst from surrounding tissues, and arterial spin labeling (ASL) MRI for measuring resting cerebral blood flow [52,61,62]. On the other hand, ACs do not demonstrate restricted diffusion on diffusion-weighted sequences (DWI), which might be a differentiating characteristic of ACs versus dermoid or epidermoid cysts [2,29,35]. MRI can detect any possible associated central nervous system (CNS) abnormalities, such as heterotopia, corpus callosum dysgenesis, or ventricular system compression [29,38], and can also contribute to the differential diagnosis of ACs from other pathological entities [22,36,38]. On CT, ACs have similar density to CSF and are not contrast-enhanced or calcified [2,22,29,35,36]. Moreover, ultrasound imaging could also detect single hypo-echoic lesions with lack of color in Doppler signals, and therefore might turn out to be useful for prenatal diagnosis [22,36,38,39]. Intracranial pressure (ICP) monitoring, single-photon emission computed tomography (SPECT), analysis of cerebral blood flow (CBF), and CT or MR cisternography are examples of other modalities that are not specific for the diagnosis of ACs but could prove to be useful for patient monitoring and screening [22,35,36,56,58]. The differential diagnosis of ACs can be wide and vary depending on the location of each lesion. A general list of the differential diagnosis can be seen in Table 3.

### 4.6. Management

Regarding the present reported patient case, a complex dilemma that required careful consideration was encountered in the course of the operation, since the cyst was not completely resected. It is worth noting that the possibility of a potential recurrence in this given scenario was notably high, given that the patient was very young and presented with a cystic lesion that affected the brainstem. Thus, the recurrence of this cystic lesion could potentially result in severe complications within this highly sensitive and critical brain region. This posed a significant hurdle in achieving the desired outcome of the surgery, requiring careful evidence-based decisions and consensus between experienced neurosurgeons. Therefore, a shunt catheter established a lasting communication channel between the cyst and the basal cisterns. The procedure in question, while rarely documented in the existing literature regarding pediatric patients [29], is underpinned by the principles of microsurgical fenestration and stereotactic cyst-ventricular drainage techniques that have previously been reported [3,63,64,65]. Therefore, the aforementioned procedure may confer benefits to patients in terms of reduced surgical risk and diminished likelihood of recurrence of the primary lesion.

The management of pediatric ACs can be a very challenging issue that raises a great deal of controversy among neurosurgeons [10]. Treatment strategies for cerebral arachnoid cysts in both pediatric and adult populations remain controversial and, despite numerous studies, there is no clear consensus on the most effective approach [10]. With regard to pediatric posterior fossa ACs, especially in infants, indications for surgical treatment and the ideal surgical option remain undetermined [66]. First-line decisions will usually consist of conservative or surgical therapeutic choices. Concerning the conservative approach, several studies support observation of the asymptomatic lesion with or without neuroimaging follow-up, due to the fact that the vast majority of ACs are indeed asymptomatic and might regress spontaneously [2,7,27,36,37,38,42,54,67]. However, it is important to take into consideration different crucial factors, such as the AC location, mass-effect phenomena, CSF flow, possible associated symptoms, the size of the AC, suspicion of a tumorous condition, and the age of the patient [3,6,22,23,35,36,37,42]. Assessing all of the possible associated parameters, indications for choosing the surgical approach can be either absolute signs, such as elevation of intracranial pressure (ICP) and progressive hydrocephalus [27,33,67], or non-specific symptomatic clinical progression [20,27,33,55].

Recent advances in microsurgery, endoscopy, and overall skull base approaches provide attractive surgical solutions and have improved treatment outcomes [68]. In general, neurosurgical options for ACs include endoscopic fenestration, cyst-peritoneal shunting, craniotomy for fenestration, or total removal of the cyst [69]. Fenestration is currently an important surgical option for ACs, offering the advantage of creating a passage (hole) between the cyst’s fluid and normal CSF-filled spaces of the cerebral ventricular system, which is made up of the ventricles, subarachnoid space, and cisterns [66]. This can be accomplished either through open neurosurgical procedures (open craniotomy) or neuroendoscopic techniques [7,19,20,24,27,29,32,35,41,42,44,53,54,58,60,67], such as endoscopic cystoventriculostomy or endoscopic cystocisternostomy [2,32,38,41,49,53,57,58]. Cyst–peritoneal shunting could also be chosen as a neurosurgical option for ACs, in order to decrease the elevated ICP and deal with any dysfunction of CSF dynamics [2,19,20,22,27,36,41,53,54,58,67]. However, a rare but serious complication after AC shunting, has recently been recognized as a shunt dependency syndrome leading to intracranial hypertension [70]. Partial microsurgical resection of the cyst wall through open craniectomy, or wide marsupialization, is still commonly preferred by neurosurgeons, whereas CT-directed stereotactic aspiration is a less frequent option [2,16,27,32,36,49,53,66,67,71,72,73]. Several recent reports describe pediatric and adult cases of ACs undergoing suboccipital craniotomy, decompression, and partial cyst excision, followed by favorable outcomes [4,74,75]. In a comprehensive series of pediatric patients with posterior fossa ACs, various neurosurgical strategies were adopted, with microsurgical fenestration being the most prevalent, with or without cyst–peritoneal shunting, while endoscopic fenestration was also commonly employed [16]. Treating ACs endoscopically can be as challenging as treating them through open surgery [66]. A recent study in children has shown that microsurgery appears to be the most effective treatment option, with fewer complications compared to shunting or endoscopy, which is attributed to an increased risk of adhesional blockage of endoscopic stomias [76]. However, another report on pediatric posterior cranial fossa ACs highlights that fenestrations also seem to carry a high risk of closure [66]. As there is no gold-standard microsurgical approach yet, the decision will be relegated to the surgeon’s strategy or anatomical challenges (total removal or suboptimal excision) [66,68]. In general, children who receive shunts tend to be younger than those who undergo fenestration, but acute surgical risks seem to be similar for both operative options [77,78]. With regard to spinal ACs, extradural cysts tend to be easily resected, whereas intradural ACs are usually fenestrated [46].

### 4.7. Complications

In addition to the different syndromes and symptoms that might occur due to the mass effect or CSF circulation obstruction, such as obstructive hydrocephalus [7,19,34], another basic complication might be the rupture of ACs, which can lead to intracystic hemorrhage, subdural hygroma, or subdural hematoma [20,35,39]. Risk factors for such complications may include head trauma and/or the larger size of a cyst [7,20,35]. Moreover, regardless of the type of the surgical procedure that might be chosen, there are different complications reported for the different neurosurgical techniques, such as subdural hematoma and fluid collection, CSF leakage, extradural hematoma, pseudomeningocele, meningitis, and possible herniation, but also shunt complications such as shunt overdrainage, shunt infection, shunt blockage, or shunt dependency, with the need for revision surgical interventions [3,15,22,23,25,27,32,49,57,60].

### 4.8. Outcome

A recent study on posterior cranial fossa ACs has shown that a better patient outcome is generally expected in only about 47% of patients. Another study highlighted that even ACs causing mild or non-specific symptoms might affect the normal function of neighboring cerebral tissue, causing neuropsychological impairment. Such cognitive deficits tend to normalize after surgery [13,15,54].

## 5. Conclusions

The present study reported an unusual case of quadriparesis caused by a large arachnoid cyst in a 30-month-old female patient. This benign malformation located in a crucial area had been complicated by severe neurological deterioration and required prompt intervention, so the patient underwent a suboccipital craniectomy in a sitting position and a microsurgical cyst excision procedure, along with the creation of a lasting communication channel between the cyst and the basal cisterns. Therefore, a couple of surgical options were actually utilized, since this patient case only permitted a partial cyst-removal and fenestration, along with the creation of a small communication channel with the basal cisterns, in order to prevent any possible future relapses. Up to the present report, postoperative complete resolution of quadriparesis secondary to a posterior cranial fossa arachnoid cyst has not previously been reported. Given the challenging presentation of this young toddler with a large posterior fossa AC affecting the brainstem, the present report indicates that the surgical combination strategy of fenestration with the placement of a drainage tube might prevent the need for a revision surgery and lead to an excellent outcome. Our primary objective is to share this intriguing and rare presentation case of a seemingly benign entity that could progress to a severe and possibly irreversible clinical outcome. Additionally, even benign conditions located in rare but crucial areas might have a significant impact on patients and require prompt intervention. It is worth noting that various treatment options have been discussed in the literature, but no consensus has been reached on the optimal approach. The present study highlights the importance of early suspicion and identification of ACs, in order to prevent the disruption of maturation during critical developmental processes and preserve important functional brain activities. Future studies might address this issue and set the guidelines for early and novel surgical strategies in children with ACs.

## Figures and Tables

**Table 1:** Brain locations for usually reported arachnoid cysts.

Localization (Frequency %)	References
**Supratentorial (42–84%)**	
- Suprasellar region	[2,7,29,31,32,35,36,37,38,39,40]
- Cerebral convexity	[2,17,29,31,32,37,38,39,40]
- Supracollicular area	[2,37,40]
- Interhemispheric fissure	[2,37,40]
- Interpeduncular fossa	[37]
- Intraventricular	[21,29,32,34,36,38,39,41]
**Infratentorial (12–46%)**	
- Cerebellopontine angle (lateral cerebellar zone)	[2,7,29,32,33,34,37,39,40]
- Vermian area	[2,37,40]
- Posterior infratentorial midline cisterns	[29]
- Supra- and Retrocerebellar space	[2,7,34]
- Prepontine area	[2,34,37]
- Intraventricular	[35,37]
- Quadrigeminal cistern	[28]

**Table 2:** Clinical features of pediatric arachnoid cysts.

Symptoms or Syndromes (Frequency %)	References
Headaches (26–60%)	[5,7,17,25,31,35,47,48,51,52,53,54,55]
Intracranial hypertension symptomatology (vomiting, visual disturbances, bradycardia, hypertension) (14–49%)	[2,3,6,7,18,24,29,31,35,36,37,39,48,54,56,57]
Hydrocephalus (18%)	[2,5,7,17,24,29,32,35,36,37,39,47,56,57,58]
Local mass-effect neurological deficits (6–32%)	[2,3,5,7,24,28,29,31,32,35,53,56,59]
Craniomegaly/macrocephaly (5–71%)	[2,3,6,17,29,31,35,36,39,51,53,54,57,60]
Endocrine disorders (5–7%)	[2,5,7,35,36,37,39,57,60]
Seizures (11–26%)	[2,3,5,6,17,25,29,31,35,36,39,47,48,53,55,56,60]
Developmental delay and cognitive deficits (5–33%)	[2,3,5,6,7,25,29,31,35,36,39,47,53,54,55,57,60]
Gait disturbance (10–33%)	[36,53,55]
Cerebellopontine angle syndrome with tinnitus, hearing loss, facial palsies, nystagmus, and vertigo (7–12%)	[29,35,39,52]
Speech disorders—aphasia (2%)	[2,59]
Spinal cord compression	[28,36]
Trigeminal neuralgia	[6]
Other less frequently reported symptoms: - Parinaud syndrome, depression - Distortion of orbit with proptosis, tic convulsif - Bobble-head doll syndrome	[6,28,35] [6,28,35] [36,39,57]
Spinal arachnoid cysts: - Pain (42%), sensory deficits (10%) - Gait instability (32%) - Bladder incontinence (7%)	[46]

**Table 3:** Differential diagnosis of arachnoid cysts.

Type of Malformation	References
Intra-axial cystic tumors such as pilocytic astrocytomas or hemangioblastomas	[35,36]
Mega cisterna magna	[34,35,38]
Dermoid and epidermoid cysts	[2,29,35]
Non-neoplastic cysts (neuroglial, neurenteric, porencephalic)	[35,38]
Blake’s pouch cysts	[34,38]
Dandy–Walker malformation	[34,36,38]
Choroid plexus cysts	[38,42]
Neurocysticercosis	[35]
Cavum veli interpositi	[42]
Craniopharyngioma	[36]

## Data Availability

The data presented in this study are all contained within the article.
